# Study of thermodynamic, mechanical, photo-catalytic, optoelectronic and thermoelectric properties of ferromagnetic CoSc_2_(S/Se)_4_ spinels for energy conversion devices: DFT + mBJ calculations

**DOI:** 10.1039/d6ra02853e

**Published:** 2026-07-02

**Authors:** Norah Salem Alsaiari, M. Aslam Khan, Mirha Arooj, N. A. Noor, Norah Alomayrah, Sohail Mumtaz, M. S. Al-Buriahi

**Affiliations:** a Department of Chemistry, College of Science, Princess Nourah bint Abdulrahman University P.O. Box 84428 Riyadh 11671 Saudi Arabia; b Institute of Physics, Khwaja Fareed University of Engineering and Information Technology Rahim Yar Khan 64200 Pakistan greatkhan17@gmail.com; c Department of Physics, University of Sargodha 40100 Sargodha Pakistan; d Department of Physics, College of Science, Princess Nourah bint Abdulrahman University P.O. Box 84428 Riyadh 11671 Saudi Arabia; e Department of Chemical and Biological Engineering, Gachon University 1342 Seongnamdaero, Sujeong-gu Seongnam-si 13120 Republic of Korea sohail.ahmed2015@gmail.com; f Department of Physics, Sakarya University Sakarya Turkey

## Abstract

With the increasing demand for materials that can perform more than one task and possess improved properties, the demand for systems that can be applied in optoelectronic and thermoelectric devices is also increasing. Spinel compounds, which are known for their ability to adapt to different structures and possess adjustable electronic and magnetic properties, have attracted much attention for further investigation. The physical properties, mechanical properties, optoelectronic properties, magnetic properties, and transport properties of the CoSc_2_(S/Se)_4_ spinel compounds are systematically examined in this research study. The *Fd*3̄*m* space group causes these compounds to have cubic-shaped crystals. Negative formation energies show that the system is thermodynamically stable, and the Born stability criteria show that it is mechanically stable. The electronic band structures and density of states (DOS) show a direct band gap, which proves that they are semiconductors. The static dielectric constants for CoSc_2_S_4_ and CoSc_2_Se_4_ are 7.4 and 8.3, respectively. Optical absorption spectra show that both compositions absorb a lot of light from the IR to the visible range, with the strongest absorption in the visible range. Adding Co ions causes ferromagnetic ordering, which is shown by the presence of strong local magnetic moments. The thermoelectric properties are examined in a 300–800 K temperature range, revealing an enhancement in both electrical and thermal conductivities as the temperature increases. Also, both compounds have high Seebeck coefficients, which range from 239 µV K^−1^ to 245 µV K^−1^. For the studied materials, the figure of merit (*ZT*) remains consistent in the said temperature range. According to the results, the CoSc_2_(S/Se)_4_ spinels can be good candidates for use in optoelectronic and thermoelectric devices.

## Abstract 2

The physical properties of the CoSc_2_(S/Se)_4_ spinels are systematically examined. The results indicate that both spinels are dynamically stable, and spin-dependent electronic properties representing a ferromagnetic semiconductor nature.

## Graphical abstract

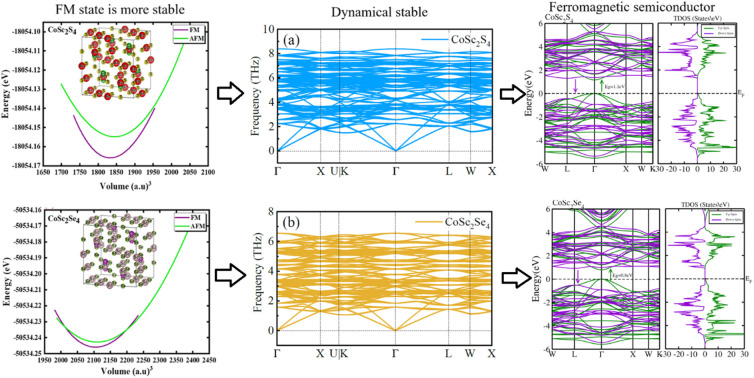

## 1. Introduction

Optoelectronics is one of the fastest-growing fields in materials science currently; it explains how light and electronic materials interact at the quantum level. It is the base for many important technologies, such as optical communication systems, biomedical instrumentation, sensor platforms, display technologies, advanced lighting and renewable energy devices.^1–5^ The latest research in optoelectronics is increasingly concentrated on identifying novel materials that exhibit superior structural stability, thermal stability, tunable electronic characteristics, robust quantum-mechanical light–matter interaction and elevated power conversion efficiency. Spinels are particularly promising candidates because of their advanced optoelectronic properties, along with the requisite mechanical and chemical strength for device integration.^6^ Recent developments on renewable energy sources, like solid-state hydrogen storage, nuclear energy and hydrogen power, has also identified them as key technologies. In renewable energy sources, spinel compounds are also potential candidates for hydrogen production. Hydrogen (H_2_) is a good energy carrier due to its renewable, environmentally friendly and highly efficient nature.^7^ Additionally, there are several hydrogen storage methods, and the hydrides have gained considerable interest mainly due to their high hydrogen storage density.^8,9^

Spinel compounds, composed as AB_2_X_4_, have a cubic crystal lattice similar to that of MgAl_2_O_4_. In this composition, the A-site is occupied by cations, which are usually alkali or alkaline-earth metals, grouped as tetrahedral sites and surrounded by four X-anions. The B-site is occupied by cations too, which are usually transition metals, grouped as octahedral sites and surrounded by six X-anions. The negatively charged X anions, for instance halides/chalcogenides, join these cation sublattices. Hence, they are bound to A and B sites, keep the overall charge neutral, and make a 3D crystal structure.^10^ Spinel compounds usually have diversity in optical properties and adjustable electronic band gaps. This is achieved through substitution or doping; hence, extensive research has been conducted on these compounds for optoelectronic applications, such as photovoltaic cells,^11,12^ laser diodes,^13^ light-emitting diodes (LEDs),^14^ photo-catalytic systems, and electroluminescent displays.^15^ In addition to their optoelectronic applications, spinels also demonstrate excellent charge transport and thermoelectric properties. They possess high electrical conductivity, low thermal conductivity, and high Seebeck coefficients, which are highly ideal for thermoelectric energy conversion.^16^

Chalcogenide spinels have gained considerable attention due to their proven thermodynamic and mechanical stability, which are essential for the fabrication of devices. It is also found in a recent study that Ln_2_MnSe_4_ (Ln = Yb, Lu) spinels may be used as thermoelectric materials. In addition, replacing Yb with Lu increases the figure of merit (*ZT*) from 0.18 to 0.79. This definitely means that the Lu-based compound has a good balance of the Seebeck coefficient, electrical conductivity, and thermal conductivity.^17^ Noor *et al.* studied HgSm_2_X_4_ (X = S, Se) spinels and proved the thermodynamic stability of the compounds based on the criteria set by them.^18^ Mustafa *et al.* also proved that HgY_2_X_4_ (X = S and Se) spinels are stable thermodynamically and mechanically, which confirmed that they could be utilized as strong functional materials. They also proved that the sulphide had a direct band gap of approximately 1.2 eV, and the selenide had a direct band gap of 0.6 eV. This indicates that these compounds can be utilized for the fabrication of optoelectronic devices that function in the infrared and near-infrared region.^19^ In a research work focusing on band gap engineering, Shahid *et al.* experimentally doped Mg_1−*x*−*y*_ Sr_*x*_Mn_*y*_ Al_2_O_4_ (*x* = 0.1–0.7 and *y* = 0.1) and Mg_1−*x*_Sr_*x*_Al_2_O_4_ (*x* = 0.1–1.0) and demonstrated that the substitution of Sr^2+^ ions at the A-site was an effective means of controlling the electronic structure. By varying the concentration of Sr^2+^ ions, they were able to control the optical band gap to a range of 4.7–5.0 eV, which was quite sensitive to the conduction and valence band edges, thereby establishing the utility of Sr^2+^ doping as a means of controlling deep-UV transparent spinel oxides.^20^ The optoelectronic properties of MgSc_2_(S/Se)_4_ compounds have been examined computationally, revealing direct band gaps of 2.3 eV for the sulfide and 1.7 eV for the selenide, along with strong optical absorption extending from the visible to the ultraviolet region, peaking at approximately 10^6^ cm^−1^ in the UV range. Their high Seebeck coefficients, *i.e.* between 220 and 235 µV K^−1^, also show that they might be used for thermoelectric applications.^21^

Keeping in mind the above-mentioned studies, the current investigation is based on the optoelectronic and thermoelectric properties of CoSc_2_(S/Se)_4_ chalcogenide spinels using DFT-based calculations. Hence, we have studied mechanical, electronic, and optical properties in order to compare and understand trends across different types of properties systematically. While chalcogenide spinels are a known class of materials, our study focuses on the specific synergistic effect of combining a transition metal (Co) with Sc, which results in a unique electronic environment that favors both magnetic order and high thermoelectric performance. After understanding these properties, we are able to link elastic behaviour to electronic band structure and optical response. This draws a better picture of how composition and crystal structure affect the overall performance of chalcogenide spinels. BoltzTrap code^22^ is used to understand the thermoelectric behavior of these compounds. Generally, our results indicate that the CoSc_2_(S/Se)_4_ spinels are promising candidates for next-generation thermoelectric and optoelectronic applications.^17^

## 2. Computational details

To investigate the mechanical, electronic, and optical responses of the CoSc_2_(S/Se)_4_ spinels, density functional theory (DFT) calculations were carried out using the WIEN2k package.^23^ The full-potential linearized augmented plane wave (FP-LAPW) method was employed in order to ensure an accurate all-electron description of the electronic structure without approximations, thereby providing a reliable basis for studying elastic and optical properties. Exchange–correlation effects were first described using the Perdew–Burke–Ernzerhof generalized gradient approximation (PBE-GGA), providing a reliable baseline for total-energy and structural optimizations. To achieve a more accurate description of the electronic spectrum, particularly the band gap and band-edge positions, the modified Becke–Johnson (mBJ) potential was subsequently applied for the calculation of the electronic band structure (EBS) and density of states (DOS), as it is known to substantially improve band gap and related electronic property predictions compared to conventional PBE-GGA.

Both ferromagnetic (FM) and antiferromagnetic (AFM) configurations of CoSc_2_S_4_ and CoSc_2_Se_4_ were considered in order to identify the magnetic ground state. For the FM configuration, all atomic sites were initialized with parallel (spin-up) alignment, resulting in a finite net magnetic moment; this moment was found to be dominated by contributions from the Co atoms, whereas the remaining atomic species contributed only weakly. In the AFM configuration, one of the two Co atoms was assigned spin-up and the other spin-down, leading to the complete cancellation of the net moment and confirming an antiferromagnetic arrangement on the Co sublattice. This particular arrangement affected the electronic structure and magnetic response of these spinels, which could be observed while looking at the energy values, *i.e.* the FM state was more stable than the AFM state. This indicated that the ferromagnetic configuration was the stable ground state.

Within the FP-LAPW framework, space is divided into non-overlapping muffin-tin (MT) spheres around the atoms and an interstitial region between them. Inside the MT spheres, the electronic wavefunctions are expanded in spherical harmonics, whereas plane waves are used in the interstitial region. The following numerical settings were adopted to ensure well-converged results. The plane-wave cut-off was controlled by setting the product of *R*_MT_ × *K*_max_ to 8, while the maximum angular momentum inside the muffin-tin spheres was limited to ℓ_max_ = 10, and the Fourier cut-off for the charge density was chosen as *G*_max_ = 18. Brillouin-zone integrations were performed using a 10 × 10 × 10 Monkhorst–Pack *k*-point grid, and self-consistent field (SCF) iterations were continued until the total energy changed by less than 10^−2^ mRy between successive cycles, providing numerically reliable and stable solutions. BoltzTraP code^22^ was used to look at the transport behaviour within the framework of the semi-classical Boltzmann transport theory. It used a constant relaxation time approximation, with *τ* = 10^−14^ s. Therefore, fix relaxation time was used for all calculations to investigate the thermoelectric performance. This method made it possible to calculate important transport coefficients and thermoelectric parameters from the electronic structure derived from DFT.

## 3. Results and discussions

### 3.1 Structural and mechanical properties

**Fig. 1:**
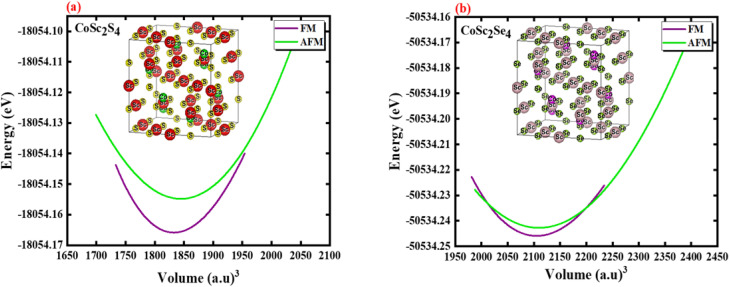
Volume optimization plots with the unit cells of the (a) CoSc_2_S_4_ and (b) CoSc_2_Se_4_ spinels in the FM and AFM spin orientations.

The crystal structure of the CoSc_2_(S/Se)_4_ spinel compounds is shown in Fig. 1. The ball-and-stick models are shown in Fig. 1(a) and (b), respectively. In the figures, the Co, Sc, and S/Se ions are denoted by red, green and yellow spheres, respectively. The figure shows the face-centered cubic (FCC) lattice with the *Fd*3̄*m* space group. Cobalt ions were located at tetrahedral positions coordinated by S/Se anions, while yttrium ions were located at octahedral positions defined by the Wyckoff positions (0.125, 0.125, 0.125), (0.5, 0, 0), and (0.25, 0.25, 0.25), with oxidation states of +3, +2, and −2 for Sc, Co, and S/Se, respectively. The unit-cell volume was relaxed to find the equilibrium lattice parameter (*a*_0_) at the ground state, where the total energy was minimum. The resulting energy–volume data were then fitted using the Birch–Murnaghan equation of state, yielding smooth equations of state and enabling the construction of the energy–volume curves shown in Fig. 1(a and b), from which bulk moduli and related elastic parameters could also be inferred.

Table 1 presents the optimized lattice constants of 10.30 Å for CoSc_2_S_4_ and 10.77 Å for CoSc_2_Se_4_, obtained at the equilibrium volumes. The substitution of sulfur by selenium led to an increase in the lattice constant, reflecting the large ionic radius of Se and the resulting expansion of the unit cell. The same trend of lattice constant by the substitution of sulfur by selenium was observed for recently reported CoY_2_X_4_ (X= S, Se) spinels.^24^ Furthermore, the volume optimization procedure was employed to examine the magnetic response by comparing ferromagnetic (FM) and antiferromagnetic (AFM) spin configurations. Fig. 1 shows that the FM configuration has less total energy, which shows that it is more thermodynamically stable than the AFM state. We figured out the formation enthalpy (Δ*H*_f_) of the compounds we studied to see how stable they were thermodynamically. The formation enthalpy was the difference in energy between the entire compound and the individual ions that make it up. It can be written as follows:1
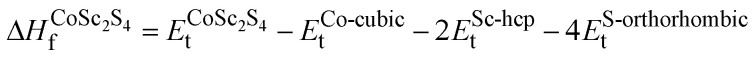
2
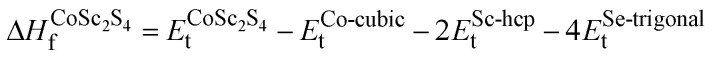
In this expression, 
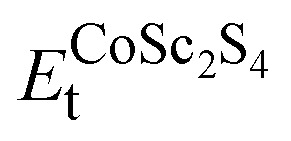
 and 
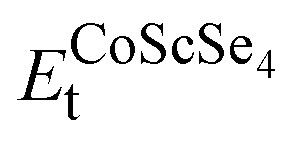
 stand for the ground-state energies per formula unit of the two spinel compounds. *E*_t_^Co-cubic^, *E*_t_^Sc-hcp^, *E*_t_^S-orthorhombic^, and *E*_t_^Se-trigonal^ stand for the energies of the elements that make up the compounds in their stable crystalline phases (cubic Co, hcp Sc, orthorhombic S, and trigonal Se). Negative Δ*H*_f_ values mean that heat is released during the formation of a compound, which is called exothermic formation. The formation enthalpies we found were −1.61 eV for CoSc_2_S_4_ and −1.39 eV for CoSc_2_Se_4_ (Table 1). This showed that both compounds were thermodynamically stable compared to the elements that made them up. CoSc_2_S_4_ released more energy than its selenide counterpart, which indicated it was more thermodynamically stable.

**Table 1:** Ground state lattice constants *a*_0_ (Å), bulk modulus *B*_0_ (GPa), enthalpy of formation Δ*H*_f_ (eV), and elastic parameters calculated for CoSc_2_S_4_ and CoSc_2_Se_4_

Composition	*a*_*0*_ (Å)	*B*_0_ (GPa)	Δ*H*_f_ (eV)	*C*_11_	*C*_12_	*C*_44_	*B*	*G*	*Y*	*ν*	*B*/*G*
CoSc_2_S_4_	10.30	72.72	−1.61	130.25	42.85	18.17	71.98	26.04	69.78	0.33	2.76
CoSc_2_Se_4_	10.77	61.91	−1.39	107.09	37.85	15.22	60.93	21.29	57.22	0.34	2.86

**Fig. 2:**
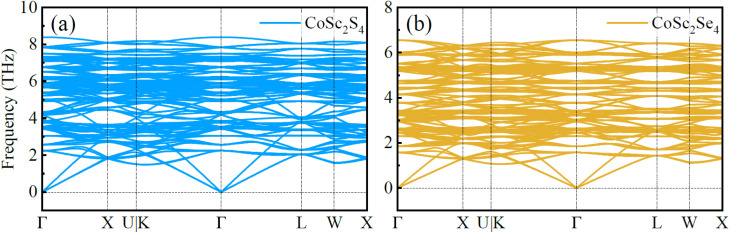
Calculated phonon dispersions for the (a) CoSc_2_S_4_ and (b) CoSc_2_Se_4_ spinels.

In order to achieve dynamic stability, the calculations of an essential aspect of the phonon dispersion relation were performed for CoSc_2_(S/Se)_4_ chalcogenide spinels, and phonon dispersion curves with symmetry lines were plotted within the Brillouin zone (refer to Fig. 2 for more information). The plot demonstrated that there were no imaginary phonon modes for positive frequencies in the Brillouin zone for CoSc_2_(S/Se)_4_, which was an indication of the compounds' dynamic stability.

To judge how well and how long devices made from a material will last, you need to know how the material reacts to stress. So, elastic stiffness constants for CoSc_2_(S/Se)_4_ were calculated to describe how they behave mechanically when stress was applied. Because spinel structures possessed cubic symmetry, their elastic behavior under applied stress could be completely characterized by only three independent elastic constants: *C*_11_, *C*_12_, and *C*_44_. The mechanical stability of these cubic systems was evaluated using the Born stability criteria, which imposed the following necessary conditions:^25^

3 *C*_44_ > 0, *C*_11_ − *C*_12_ > 0, *C*_11_ + 2*C*_12_ > 0

The calculated elastic constants, *C*_11_, *C*_12_, and *C*_44_, of the studied spinel compounds met the criteria for mechanical stability, as mentioned above (Table 1). The calculated elastic constants (*C*_*ij*_) were also employed to calculate the macroscopic mechanical properties, namely Young's modulus (*Y*), bulk modulus (*B*_0_), and shear modulus (*G*), using the following equations:^26^

4 *B*_0_ = (*C*_11_ + 2*C*_12_)/3

The total (polycrystalline) shear modulus (*G*) was then computed as the arithmetic mean of the Voigt shear bound (*G*_V) and the Reuss shear bound (*G*_R), giving a more accurate approximation of the total resistance to shear deformation in the isotropic aggregate. This is equivalent to the Voigt–Reuss–Hill approximation, where the shear modulus *G* is given by

5 *G*_R_ = [5*C*_44_(*C*_11_ − *C*_12_)]/[4*C*_44_ + 3(*C*_11_ − *C*_12_)] *G*_v_ = (3*C*_44_ + *C*_11_ − *C*_12_)/5

6 *G* = (*G*_R_ + *G*_v_)/2

*B*_0_ and *G* values are further utilised to calculate *Y* through this equation:

7 *Y* = (9*B*_0_*G*)/(3*B*_0_ + *G*)

The bulk modulus, derived from volume optimization using the Birch–Murnaghan equation of state, fitted the energy–volume curve very well and matched the calculated value from the elastic constants. The relatively reduced elastic moduli with the substitution of sulfur by selenium implied that CoSc_2_S_4_ was stiffer than CoSc_2_Se_4_. The reduced stiffness in the selenide material could be attributed to the reduced bonding interactions, which could be ascribed to the reduced electronegativity difference between selenium and the cationic species.^27^

Poisson's ratio (*ν*) and Pugh's ratio (*B*_0_/*G*) were also used to determine the ductile or brittle character of these compounds. In cubic crystals, the criteria for ductility are given by *ν* > 0.26 and *B*_0_/*G* > 1.75, while for brittleness, the values are below these thresholds.^28,29^ The calculated values for both compounds met the criteria for ductility (Table 1). Another criterion for determining the ductile or brittle character of a compound is the Cauchy pressure, which is given by *C*_12_ − *C*_44_. A positive value indicates a ductile compound, while a negative value indicates a brittle compound.^30^ The positive Cauchy pressure calculated for these spinel compounds (Table 1) also supports their ductile nature.

**Fig. 3:**
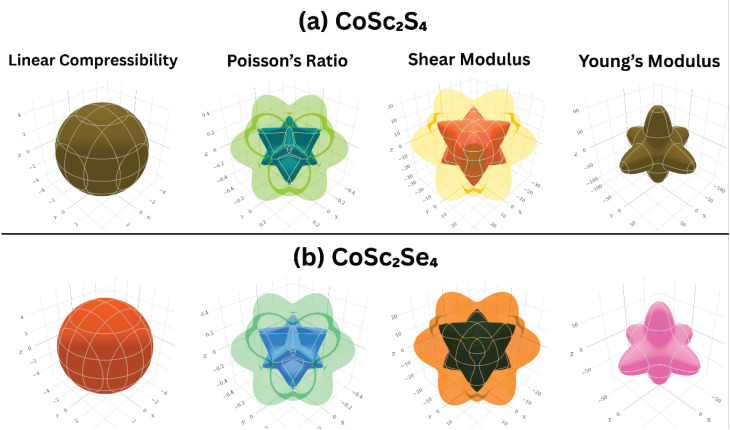
3D representations of linear compressibility, Poisson's ratio, Shear modulus and Young's modulus for the (a) CoSc_2_S_4_ and (b) CoSc_2_Se_4_ spinels.

Three-dimensional surface plots of the Young's modulus (*Y*), bulk modulus (*B*), shear modulus (*G*), Poisson's ratio (*ν*), and Pugh's ratio (*B*/*G*) were generated to analyze the elastic anisotropy of these spinel compounds (Fig. 3). The orientation-dependent surface plots provided a clear indication of how the elastic properties varied with crystal direction, thereby providing information on the anisotropic nature of the elastic properties. The bulk modulus surface plot was close to being spherical, suggesting that the material's resistance to uniform compression was almost isotropic. However, the large anisotropy in the three-dimensional surface plots of *G*, *Y*, and *ν* clearly indicated the material's elastic anisotropy in CoSc_2_(S/Se)_4_.

The anisotropic properties of CoSc_2_(S/Se)_4_ could also be established by the large directional variations in its mechanical properties, as indicated by the large differences between the maximum and minimum values of the shear modulus (*G*) and Young's modulus (*Y*) along various crystallographic directions. The results of the analysis of the anisotropy factors, elastic constants, and calculated moduli established that CoSc_2_(S/Se)_4_ was mechanically stable, highly elastically anisotropic, and ductile. These properties together make these compounds very attractive for optoelectronic and energy-related device applications. The isotropic linear compressibility (*β*) of the CoSc_2_(S/Se)_4_ spinel compounds could be seen from the values of *β*_min and *β*_max, which were almost equal in all the crystallographic directions. This showed that the compounds were equally compressible in all directions under the applied pressure, which is a common feature of cubic crystals.

### 3.2 Electronic properties

**Fig. 4:**
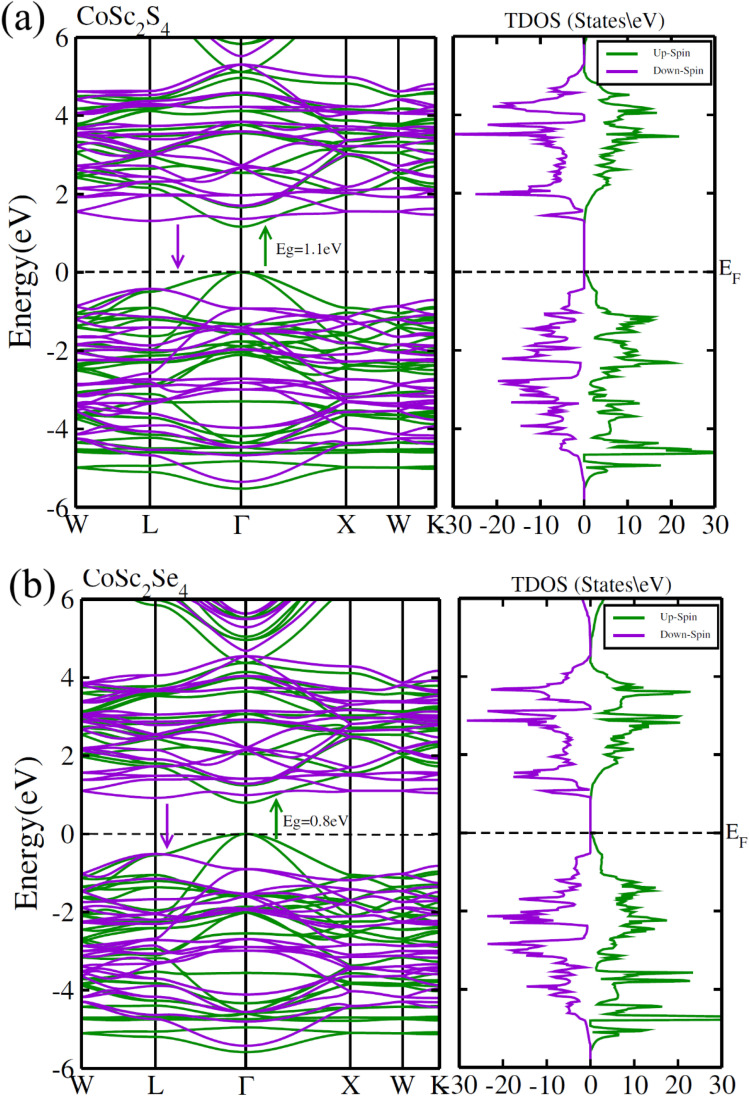
Electronic band structures and total DOS plots of the (a) CoSc_2_S_4_ and (b) CoSc_2_Se_4_ spinels, computed with the mBJ potentials.

The spin-polarized density of states (DOS) and electronic band structure (EBS) of CoSc_2_(S/Se)_4_ were studied in depth in order to figure out electronic conductivity. Hence, energy dispersion and state distribution within a window of ±6 eV around the Fermi level (*E*_F_) were especially observed in these calculations. This provided a clearer picture of band edges and states that were very important for charge transport characteristics. Fig. 4a and b represent band structures for the CoSc_2_S_4_ and CoSc_2_Se_4_ spinel, respectively. The Fermi level was in the middle of the conduction band (CB) and the valence band (VB) in both cases. This proved that these spinels were semiconductors. Also, both compounds had direct band gaps because the highest point in the valence band and the lowest point in the conduction band were at the same point in the *Γ* high-symmetry point for both spin channels. For CoSc_2_S_4_, the spin-up channel had a direct band gap of 1.1 eV, with the valence band maximum (VBM) and the conduction band minimum (CBM) lying at the *Γ* point. For the spin-down channel, a relatively large direct band gap (L–L) was also observed (1.8 eV) (see Table 2). Similarly, CoSc_2_Se_4_ had direct band gaps of 0.8 eV (Γ–Γ) and 1.2 eV (L–L), respectively, for the spin-up and spin-down channels. The gap of the S-based compound suggested greater electronic conductivity and greater absorption of visible light than those of the Se-based compound. Similar trends of the direct band gap having the same point in the *Γ* high-symmetry point for both spin channels are reported in recent studies on MnSc_2_S_4_ and MnSc_2_Se_4_ spinels.^31^ The calculated band structures clearly showed this difference, and Fig. 3 shows it in a nutshell. Owing to the spin-dependent nature of the band gaps, the electronic states underwent exchange splitting, indicating that the materials thus were magnetic semiconductors. Moreover, the direct gaps indicated that the optical transitions occurred without the assistance of phonons, which made them potential candidates for optoelectronic applications.

**Table 2:** Calculated band gap values for the spin up (↑) and spin down (↓) channels, their nature, and VBM and CBM positions for CoSc_2_S_4_ and CoSc_2_Se_4_

Compound	Spin channel	Band gap (eV)	Nature	VBM position	CBM position
CoSc_2_S_4_	↑	1.1	Direct	Γ	Γ
CoSc_2_S_4_	↓	1.8	Direct	L	L
CoSc_2_Se_4_	↑	0.8	Direct	Γ	Γ
CoSc_2_Se_4_	↓	1.2	Direct	L	L

The selenium-based compound had a relatively small band gap because of two main things: (i) the small difference in electronegativity between Se and the cations around it and (ii) the extra electronic states that selenium adds.^32^ The small difference in electronegativity made the covalent character stronger and the electronic states more spread out, which made them less likely to be found around individual atomic sites. The extra states that came from Se also created extra bands near the band edges. This shifted both the valence and conduction bands closer to the Fermi level and made the overall band wider, which lowered the fundamental band gap.^33^

Total density of states (TDOS) and partial density of states (PDOS) show how constituents of a compound affect the net electronic structure of the compound. Using GGA and combined with mBJ methods, Fig. 4 represents TDOS for both CoSc_2_S_4_ and CoSc_2_Se_4_ spinels. The existence of the band gap around the Fermi level clearly showed that these spinels were semiconductors in nature. The GGA + mBJ method produced larger band gaps compared to using the GGA. The gap in CoSc_2_S_4_ was 1.1 eV, which was more than the gap in CoSc_2_Se_4_, which was 0.8 eV. This improvement showed that the systematic underestimation that happens in standard GGA calculations was fixed, which was in line with the mBJ potential's well-known accuracy. CoSc_2_Se_4_ always had a smaller band gap than CoSc_2_S_4_, no matter how you figure it out. This showed how Se could control the electronic structure in two ways: its extra valence states added extra bands near the band edges, and its lower electronegativity compared with those of the surrounding cations made covalency and state delocalisation stronger. Together, these effects compressed the separation between valence- and conduction-band edges, leading to a noticeable reduction in the band gap.^34,35^

**Fig. 5:**
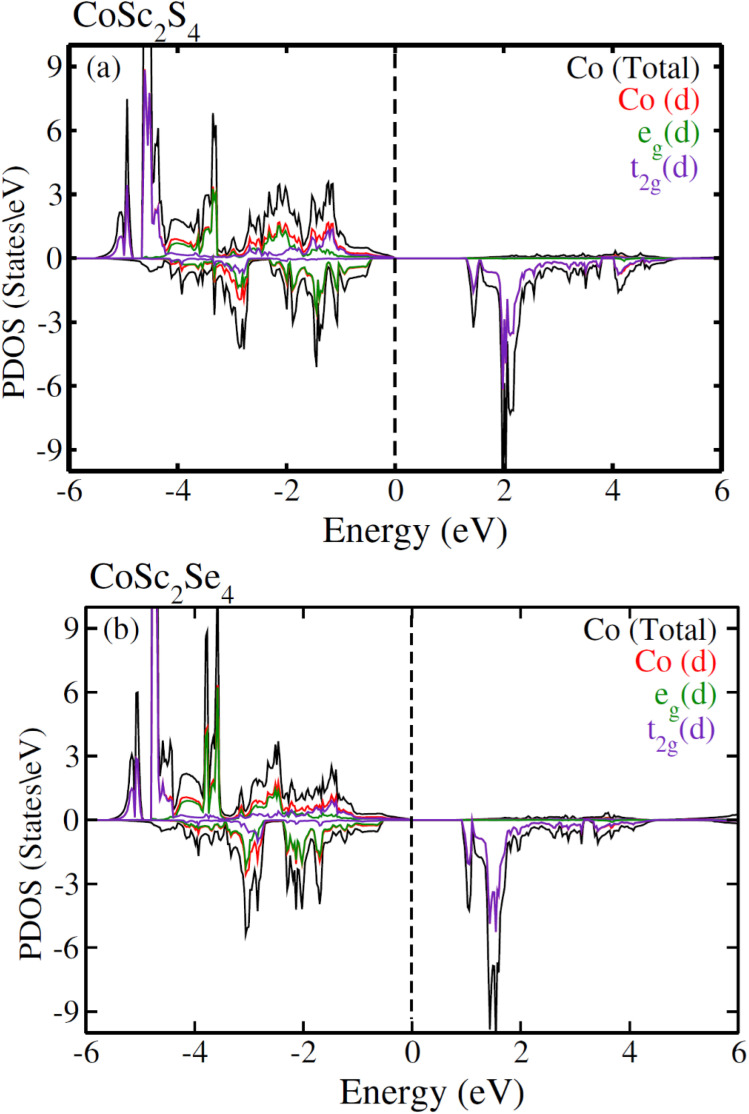
Partial DOS plots of the (a) CoSc_2_S_4_ and (b) CoSc_2_Se_4_ spinels in the spin-up (↑) and spin-down (↓) configurations, computed with the mBJ potentials.

The GGA + mBJ approach was employed to determine the PDOS of CoSc_2_S_4_ and CoSc_2_Se_4_, as depicted in Fig. 5. For both materials, the t_2g_ subset of the Co 3d orbitals was found to dominate the valence band just below the Fermi level. This implied that Co atoms primarily dictated the bonding character at the edge of the valence band. The Co 3d-derived states that contributed to the conduction band were relatively more distributed in energy and had a distinctly lower spectral intensity compared to several other contributing states. This suggests a reduced density of Co 3d states near the conduction band, which could significantly affect carrier mobility and the overall transport character of these spinel materials. The Co d-states in CoSc_2_S_4_ were relatively more localized and sharper at the edge of the valence band. However, the CoSc_2_Se_4_ compound had less intense and broader features due to the relatively small electronegativity difference between Se and Co, which favored the delocalization of the electronic states.

### 3.3 Optical properties

Optical properties are significant to comprehend the effect of light interaction with matter and the movement of carriers, which further explains the workings of optoelectronic systems. For the fabrication and analysis of semiconductor materials for photodetectors, solar cells, and light-emitting diodes, it is significant to comprehend the mentioned phenomena. The current research work comprehensively analyzed the optical properties of both compounds, such as dielectric function (*ε*), absorption coefficient (*α*), complex refractive index (*ñ*), and reflectivity (*R*). Calculations were carried out while taking into account the photon energy spectrum ranging from 0 to 8 eV, adjudicating their entire optical behavior.

**Fig. 6:**
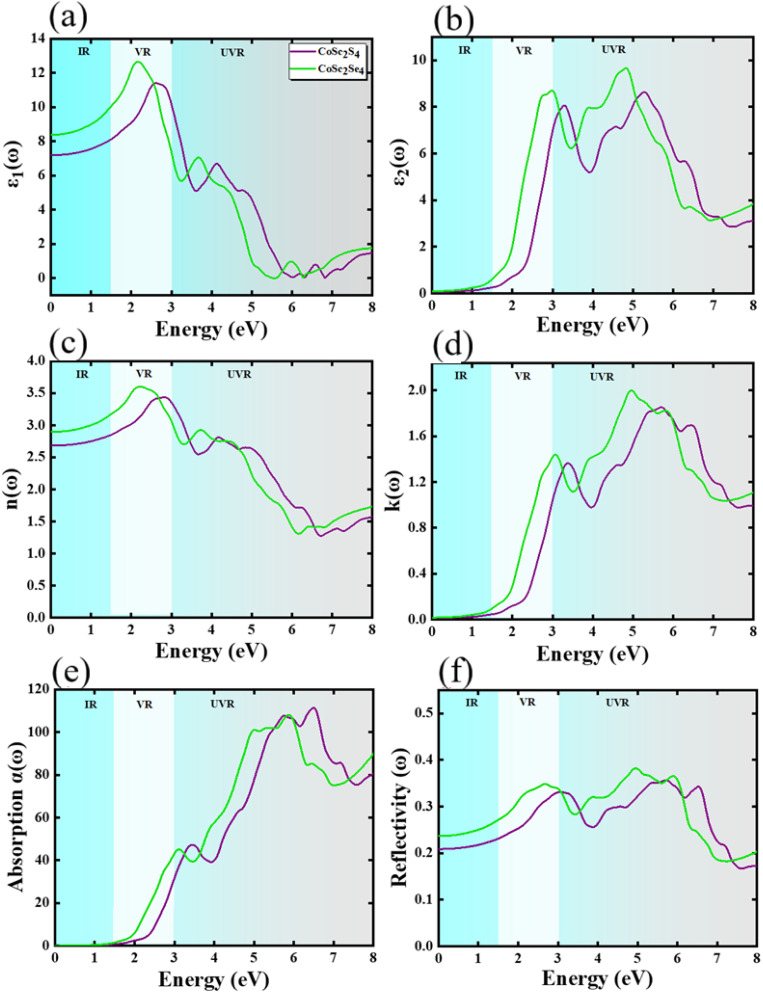
Calculated (a) real part (*ε*_1_(*ω*)), (b) imaginary part (*ε*_2_(*ω*)), (c) refractive index (*n*(*ω*)), (d) extinction co-efficient (*k*(*ω*)), (e) absorption (*α*(*ω*)), and (f) reflectivity (*R*(*ω*)) of the CoSc_2_S_4_ and CoSc_2_Se_4_ spinels.

First, we calculated the dielectric constant, which gives us an idea of how the material responds to electromagnetic radiation. The real part, *ε*_1_, gives us an idea of how the material polarizes when an electric field is applied, and the imaginary part, *ε*_2_, gives us an idea of how the material loses energy. Fig. 6(a) shows the graph for *ε*_1_, which indicates that the static dielectric constant for CoSc_2_S_4_ is 7.4 and for CoSc_2_Se_4_ is 8.2. This indicated that the dielectric constant and band gap energy were inversely proportional to each other, as *ε*_1_ was roughly inversely proportional to the band gap energy. Also, *ε*_1_ decreased at relatively high optical frequencies, where dipolar polarization became ineffective. The imaginary part (*ε*_2_) remained positive for the entire range of frequencies.

The real and imaginary parts are related through the Kramers–Kronig expressions:^36^8
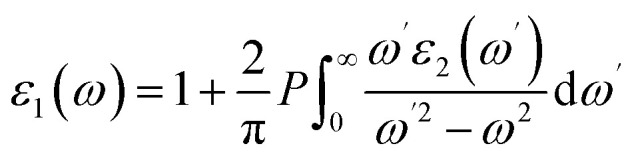


The calculation of *ε*_2_(*ω*) involves the following expression:9
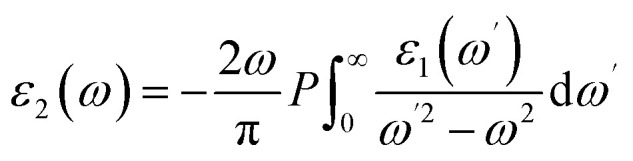


The calculated *ε*_2_ spectra are shown in Fig. 6(b). When the photon energy was above the band gap (*E*_g_) for both spin channels, there were prominent peaks. This was because there were large dielectric losses due to strong interband transitions. The peaks occurred at 2.8 eV for CoSc_2_S_4_ and 2.2 eV for CoSc_2_Se_4_. These were the energies at which the maximum amount of light was absorbed.

Another key optical property was the complex refractive index, *ñ* = *n* + *ik*. The real part (*n*) indicates how much slower the waves propagate in the material compared to the speed in a vacuum. The imaginary part, the extinction coefficient (*k*), indicates the loss of energy of the electromagnetic waves while propagating in space due to scattering and absorption.^37^ The *n* and *k* spectra, which were calculated, are depicted in Fig. 6(c) and (d), respectively. The static refractive index (*n*(0)) was approximately 2.7 for CoSc_2_S_4_ and 2.9 for CoSc_2_Se_4_. The refractive index increased with increasing photon energy until it reached approximately 3 eV, beyond which it decreased slowly. The extinction coefficient (*k*) also had strong maxima, with a strong peak occurring at 3.1 eV for the sulfide and a peak at 3.5 eV for the selenide. These high-energy features signaled intense optical absorption, arising from interband electronic transitions in the deep-visible region, and they underscored the sensitivity of the high-frequency optical response to chalcogen substitution. The refractive index and extinction coefficient relate to the dielectric constants *via n* = √[(*ε*_1_ + √(*ε*_1_^2^ + *ε*_2_^2^))/2] and *k* = √[(−*ε*_1_ + √(*ε*_1_^2^ + *ε*_2_^2^))/2]. Consequently, the *n* spectrum closely followed the *ε*_1_ trend, while *k* exhibited a mirrored response relative to *ε*_2_.

Interband electronic transitions cause semiconductors to absorb light. Fig. 6(e) shows the absorption coefficient (*α*), which measures this process. When the energy of a photon meets or exceeds the band gap, absorption starts. This process speeds up quickly as the incoming electromagnetic energy goes above this point. Both compounds took in light from the visible spectrum, but they took in the most light from the visible region. Reflectivity (*R*(*ω*)) measures how much electromagnetic radiation is lost when it is reflected. This is an important factor for optoelectronic materials because it affects how well they work. Fig. 6(f) shows the reflectivity spectra for both materials. The reflectivity values were 0.21 for CoSc_2_S_4_ and 0.23 for CoSc_2_Se_4_ when the photon energy was zero. As the photon energy increased, the reflectivity values reached their highest points of 0.33 for CoSc_2_S_4_ and 0.35 for CoSc_2_Se_4_ in the visible region.

### 3.4 Magnetic properties

We have looked at the magnetic properties of CoSc_2_S_4_ and CoSc_2_Se_4_ using crystal field effects, exchange interactions, and other related factors. Spin-resolved band structure analysis showed exchange splitting, which is when the band gaps between spin channels are different because of interactions between Co and the chalcogen (S/Se) atoms. In the octahedral coordination environment, the chalcogen atoms divide the Co 3d orbitals into five different energy levels: a lower-energy degenerate triplet (t_2g_) (which includes d_*xy*_, d_*yz*_, and d_*zx*_) and a higher-energy doublet (e_g_) (which includes d_*x*^2−^y^2^_ and d_z^2^_). The crystal field splitting energy (ΔCF) is the difference in energy between the bonding (e_g_) and antibonding (t_2g_) states. Cobalt ions' electronic energy levels slightly alter in crystal environments. The d_z^2^_ orbital showed more energy than the d_*xy*_ and d_*yz*_ orbitals, which partly reversed the degeneracy; hence, it was in accordance with Hund's rule. It also regulated the distribution and interactions of d-electrons with other atoms. The ferromagnetic behaviour seen was mostly because the sulphur or selenium p orbitals mixed with the cobalt 3d orbitals. This interaction is very important for figuring out what the material's electronic and magnetic properties are.

The exchange interaction caused the t_2g_ states to move to lower energy levels. Because of this, the six electrons in the cobalt 3d shell prefer to be in these lower-energy bonding states, which are made more stable by the crystal field and p–d orbital hybridisation working together. This arrangement of electrons lowers the system's total energy and sets the ground-state spin configuration, which causes the material to behave magnetically. Δ(Pd), the exchange-splitting energy, gives a numerical value for this effect. The relative positions of the anionic p-state valence-band edges in the spin-down and spin-up channels decide it. This is shown by equation Δ(Pd) = *E*^↓^_V_ − *E*^↑^_V_. You can get this value directly from the calculated band structure or from different parts of the spin-resolved density of states. The negative values of Δ(Pd) showed that the spin-down configuration was more energetically favourable than the spin-up configuration. This shows the intrinsic energy imbalance that spin-dependent interactions cause, which is a defining feature of spin-polarized materials.

Current investigation elucidated the magnetic properties of CoSc_2_S_4_ and CoSc_2_Se_4_ through the incorporation of electronic state transitions, crystal field effects (written as ΔCF for both spin-up and spin-down configurations) and exchange energies. For exchange splitting energy (Δ(d)), dominant d-state peaks were observed in the spin-down (↓) and spin-up (↑) channels, as observed in Table 2.

A significant difference between peak energies indicates a substantial exchange interaction that affects d orbitals. Larger values of Δ(d) were observed compared with those of ΔCF, which clearly indicated that ferromagnetic exchange was more important than magnetic obstruction in the compounds. The magnetic properties of the compounds arose from a subtle equilibrium between the crystal field effects, which disrupted and rearranged the d-orbital exchange interactions and energy levels, causing spin alignments.

Exchange energy emerged due to p–d coupling between the Co 3d orbitals and the S/Se p orbitals. The negative values of the exchange energy lowered the total energy of the system, which was a major cause of stable ferromagnetism. The following equations can be used to find the exchange constants, *i.e. N*_0_*α* and *N*_0_*β*, which represent the strengths of p–d and s–d couplings.10
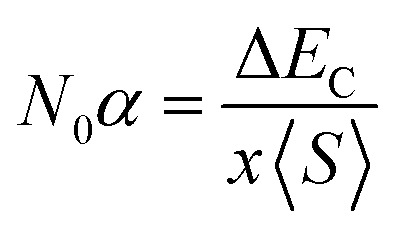
11
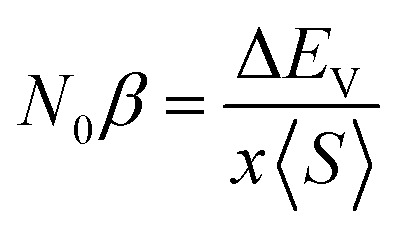


The above-mentioned relations include an average magnetic moment and a Co contribution factor, *i.e.* “*x”* as major parameters. The −ve values near the valence band edge in the spin-down channel showed that the system lowered its total energy due to the parallel alignment of neighboring spins (see Table 3). Spin-dependent band shift stabilized the charge carrier movement in the spin-down configuration, thereby promoting ferromagnetic ordering through kinetic-energy gain associated with charge transportation between mixed valence Co sites.

**Table 3:** Calculated crystal field energy, exchange energies and constants calculated for CoSc_2_S_4_ and CoSc_2_Se_4_

	ΔCF	Δ_*x*_ (d)	Δ_*x*_ (pd)	*N*_0_ *α*	*N*_0_ *β*
CoSc_2_S_4_	1.8	5.4	−0.5	0.29	−0.284
CoSc_2_Se_4_	1.4	4.9	−0.7	0.23	−0.413

The separation of the conduction and valence band edges was studied in order to find the reason for the ferromagnetic (FM) behaviour. Energy differences provide the cause of ferromagnetism. Taking into account inverse exchange interactions, the −ve exchange energy in the spin-down channel suggested the ferromagnetic behavior of the compound. Further, this made the spin-down configuration more energetically favourable than other magnetic arrangements and kept the ferromagnetic ground state stable. Spin-oriented volume optimization confirmed the ferromagnetic (FM) ground state of the compounds. We figured out the net, interstitial and local magnetic moments for both compounds in order to find the strength of magnetization (see Table 4). For both compounds, the net magnetic moment was 3 µ_B_, whereas Co-ions contributed 2.4936 µ_B_ to CoSc_2_S_4_ and 2.4621 µ_B_ to CoSc_2_Se_4_ spinel. Major contribution came from the unpaired electrons of the Co 3d orbitals, as shown by the spin-polarized partial density of states, which displayed occupied spin-up states at the valence band edge. However, Sc and S/Se atoms did not influence much, whereas the interstitial region contributed to the magnetic moment.

**Table 4:** Total, interstitial and local magnetic moments (in Bohr magnetons) calculated for CoSc_2_S_4_ and CoSc_2_Se_4_

	Total (*µ*_B_)	Interstitial (*µ*_B_)	Co (*µ*_B_)	Sc (*µ*_B_)	X (*µ*_B_)
CoSc_2_S_4_	3.0000	0.5397	2.4936	0.0528	0.0328
CoSc_2_Se_4_	3.0000	0.5618	2.4631	0.0627	0.0327

### 3.5 Thermoelectric properties

Thermoelectric (TE) materials are a good option for energy harvesting because they can directly turn heat into electricity. They became very popular due to their efficiency in traditional power generation. Additionally, such thermoelectric materials are promising autonomous energy-harvesting components in device architectures for their durability and sustainability, where they can continuously convert waste heat into useful power without the need for external fuel. Due to these reasons, this investigation used the BoltzTraP code to compute the transport properties of CoSc_2_S_4_ and CoSc_2_Se_4_. We examined some important transport parameters, such as electronic thermal conductivity (*κ*_e_/*τ*), electrical conductivity (*σ*/*τ*) and the Seebeck coefficient (*S*), within a broad temperature range from 200 K to 800 K. Because of this wide range, the assessment of their thermoelectric response from cryogenic to high-temperature regimes was possible and provided insight into how carrier transport evolved under practical operating conditions. Because different spin configurations provide diverse responses for these properties, transport parameters were computed using the following relations.^38^12
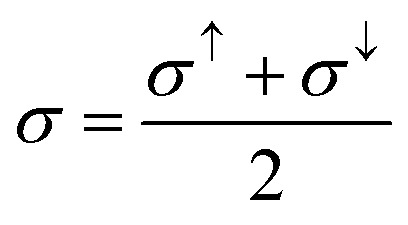
13
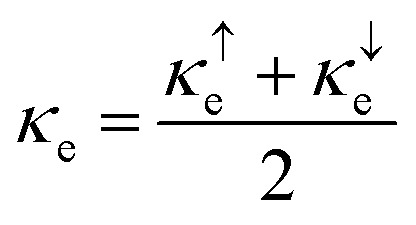
14
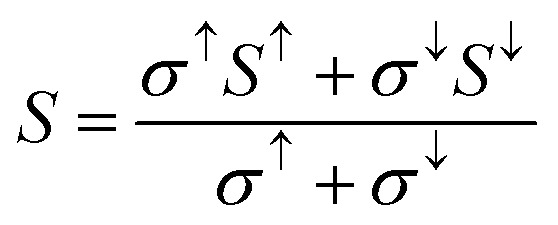


**Fig. 7:**
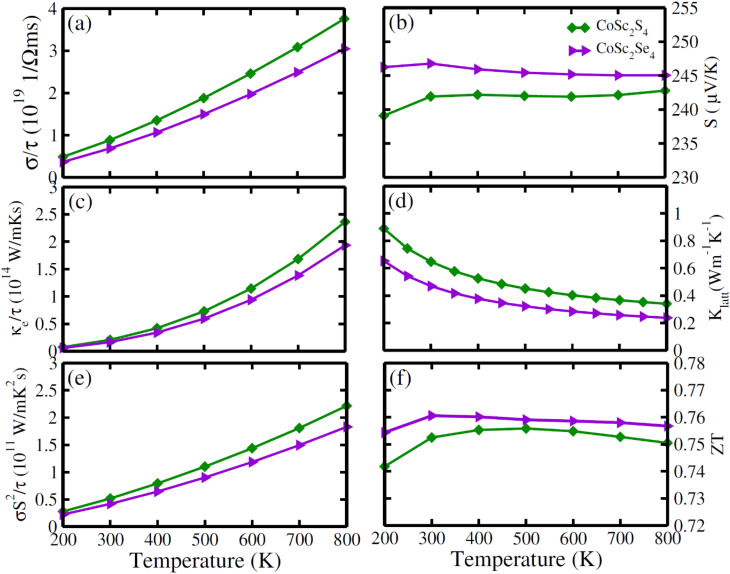
Calculated (a) electrical conductivity (*σ*/*τ*), (b) Seebeck coefficient (*S*), (c) electronic thermal conductivity (*k*_e_/*τ*), (d) lattice thermal conductivity (*k*_latt_), (e) power factor and (f) figure of merit (*ZT*) plots against temperature for the CoSc_2_S_4_ and CoSc_2_Se_4_ spinels.

Fig. 7(a) shows how much electrical conductivity there is per relaxation time (*τ*). As the temperature increased, the conductivity increased correspondingly, in a clean, straight-up fashion. This is what is expected for semiconducting materials that are only partially insulating. As you raise the temperature of these materials, the charge carriers become excited from the valence band to the conduction band, increasing in number and facilitating the flow of current. For CoSc_2_S_4_, the conductivity per relaxation time, *σ*/*τ*, was 0.5 × 10^19^ (Ω ms)^−1^ at 200 K, while for CoSc_2_Se_4_, it was 0.4 × 10^19^ (Ω ms)^−1^. Therefore, at room temperature, the conductivity of the sulfur compound was comparable to that of the selenide compound, with a slight advantage for the sulfide compound. However, as the temperature was increased to 800 K, these values shot up to 0.38 × 10^19^ (Ω ms)^−1^ for the sulfide compound and 0.30 × 10^19^ (Ω ms)^−1^ for the selenide compound.

The total thermal conductivity (*κ*) is the sum of two components: *κ* = *κ*_e_ + *κ*_l_. The *κ*_e_ component is the part that arises from the heat transport by moving electrons, while the *κ*_l_ component is the lattice contribution due to phonons propagating through the crystal lattice. In Fig. 7(c), the behavior of *κ*_e_/*τ* is shown, and it increases, just like *σ*/*τ*. At 100 K, *κ*_e_/*τ* was 0.2 × 10^14^ W m^−1^ K^−1^ s for both CoSc_2_S_4_ and CoSc_2_Se_4_, reaching their highest values of 2.4 × 10^14^ and 1.9 × 10^14^ W m^−1^ K s at 800 K. The lattice thermal conductivity, *κ*_l_, was also calculated independently using the Slack equation,^39^ and the data are plotted in Fig. 7(d). Over the temperature range, *κ*_l_ decreased monotonically from approximately 0.38 W m^−1^ K^−1^ at 200 K to 0.22 W m^−1^ K^−1^ at 800 K for both CoSc_2_S_4_ and CoSc_2_Se_4_. This is because, at high temperatures, phonon–phonon scattering becomes more prominent, suppressing lattice-mediated heat transport. More importantly, *κ*_l_ remained much lower than the electronic part, *κ*_e_, over the entire temperature range, suggesting that the transport of heat was dominated by charge carriers rather than phonons. As a result, the overall thermoelectric figure of merit, *ZT*, was only weakly affected by the lattice part.

When a thermoelectric material encounters a temperature gradient, charge carriers diffuse from the hot to the cold side of the material, leading to an electrochemical potential and measurable voltage build-up (between terminals); this effect can be assessed using the Seebeck coefficient (*S*). The magnitude of *S* is usually calculated by the following relationship:^40^15
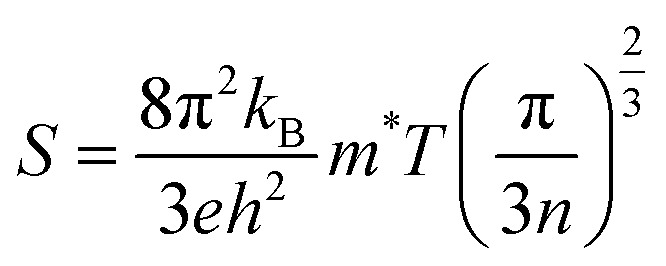
Here, *k*_B_, *h*, and *e* represent the Boltzmann constant, Planck's constant, and charge, respectively. Parameter m* stands for the carrier's effective mass. Parameter *n* is called the carrier concentration, and *T* is the absolute temperature.

Fig. 7(b) shows how the Seebeck coefficient (*S*) changes with temperature for CoSc_2_S_4_ and CoSc_2_Se_4_. This shows how their thermoelectric response changes as the temperature changes. Both compounds had Seebeck coefficients that were always high, staying above about 240 µV K^−1^ to 250 µV K^−1^ the whole time. This indicated that they could easily change thermoelectric energy into other forms of energy and had a lot of thermopower. Because *S* was always positive at all temperatures, it showed that holes were the main charge carriers. This implied that both materials were capable of conducting electricity independently. In the case of CoSc_2_S_4_, the Seebeck coefficient was approximately 235 µV K^−1^ at 300 K, and then, it gradually decreased with a mild trend towards relatively low values, reaching a value of 230.6 µV K^−1^ at 800 K. This mild trend was a result of the competing effects of the increase in carrier concentration and the widening of the carrier energy distribution with temperature. The mild decrease in the Seebeck coefficient of CoSc_2_S_4_ was primarily caused by the thermally activated increase in carrier concentration, which, consistent with the conventional inverse proportionality between the Seebeck coefficient and carrier concentration in both degenerate and non-degenerate semiconductors, led to a decrease in the Seebeck coefficient. On the other hand, CoSc_2_Se_4_ exhibited high thermal stability, with a Seebeck coefficient that varied only slightly from 239 µV K^−1^ at 300 K to 248 µV K^−1^ at 800 K.

Power factor (PF) was calculated in order to further investigate the thermoelectric potential for the CoSc_2_S_4_ and CoSc_2_Se_4_ compounds. Fig. 7(e) shows the calculated PF, which clearly rises with temperature because the electrical conductivity also rises at the same time. The PF values for CoSc_2_S_4_ and CoSc_2_Se_4_ at 300 K were 0.3 × 10^11^ W m^−1^ K^−2^ s and 0.2 × 10^11^ W m^−1^ K^−2^ s, respectively. At 800 K, the power factor afforded the highest values of 2.2 × 10^11^ and 1.8 × 10^11^ W m^−1^ K^−2^ s.

We used the figure of merit (*ZT*) to measure the general thermoelectric efficiency of the compounds, *i.e.* CoSc_2_S_4_ and CoSc_2_Se_4_. Figure of merit is usually defined as follows: *ZT* = *S*^2^*σT*/*κ*, where *S* is the Seebeck coefficient, *σ* is the electrical conductivity, and *κ* is the thermal conductivity (*κ* = *κ*_e_+ *κ*_latt_), whereas *T* is the absolute temperature. Fig. 7(f) represents *ZT* for both CoSc_2_S_4_ and CoSc_2_Se_4_ compounds with respect to the temperature, which is actually a straight line over a certain temperature range. This steady improvement indicated that both materials had stable and progressively better thermoelectric performances from near room temperature to relatively high operating temperatures. This shows that they are good for energy conversion applications that work at medium to high temperatures.

### 3.6 Thermodynamic and photo-catalytic properties

**Fig. 8:**
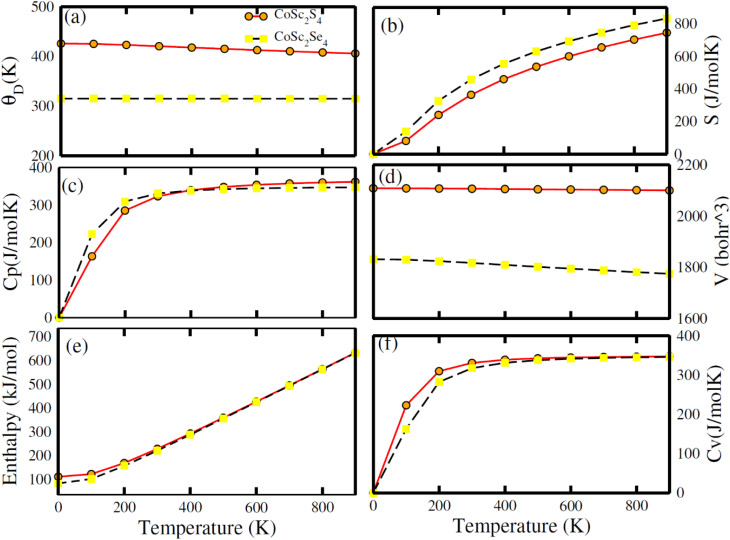
Calculated values of the (a) Debye temperature (*θ*_D_), (b) entropy (*S*), (c) specific heat (*C*_p_), (d) volume (*V*), (e) enthalpy and (f) *C*_p_ for the CoSc_2_S_4_ and CoSc_2_Se_4_ spinels.

Heat capacity (*C*_P_), being one of the basic thermodynamic properties, is often explored in the field of materials science. Although thermodynamically complex, heat capacity provides important information about the thermodynamics of the material and can be expressed in various ways.^41^ The change in entropy with temperature and pressure is closely associated with the Debye temperature (*θ*_D_), an essential characteristic of the vibrational spectra and mechanical properties of solids. As depicted in Fig. 8(a), the Debye temperature (*θ*_D_) remains nearly constant at low (cryogenic) temperatures but starts decreasing almost linearly at high temperatures, *i.e.* beyond 415 K for CoSc_2_S_4_ and 310 K for CoSc_2_Se_4_. The decreasing trend of the Debye temperature was attributed to the phonon softening and anharmonicity at high temperatures.^42^

Of all the various thermodynamic parameters, entropy (*S*) is a definitive marker of configurational disorder and phonon populations in the crystal lattice. As illustrated in Fig. 8(b), with increasing temperature, entropy increases, tending to be 0 at absolute zero temperature, in accordance with thermodynamics 3rd law.^43^ At low temperatures, entropy increased very sharply due to rapid phonon populations, but at high temperatures, it gradually saturated due to saturated vibrational modes. At 200 K, entropy values were found to be 300 J mol^−1^ K^−1^ for CoSc_2_S_4_ and 380 J mol^−1^ K^−1^ for CoSc_2_Se_4_, as illustrated in Fig. 8(b). The continuous increase in entropy from sulfide to selenide reflected the effect of increased atomic mass and vibrational modes in the heavier spinel compound.

As shown in Fig. 8(c), the constant-pressure heat capacity (*C*_P_) has a strong temperature dependence at low temperatures and becomes less pronounced as the temperature increases. Among the two compounds, CoSc_2_S_4_ had the highest (*C*_P_) value, followed by CoSc_2_Se_4_. This trend could be attributed to the rise in the atomic mass of the chalcogen components (S < Se), which influenced the lattice vibration and, subsequently, the heat capacity. The constant-volume heat capacity (*C*_V_) provides additional information regarding the solid-state transition, electronic band structure, and phonon properties.^44^ The temperature dependence of *C*_V_ is shown in Fig. 8(f), which has a strong temperature dependence below 300 K and a weak temperature dependence at high temperatures. As expected from the Dulong–Petit law, *C*_V_ became temperature-independent at approximately 800 K. At any given temperature, *C*_V_ had an S < Se trend, indicating that the lattice vibration was mass-dependent. A detailed understanding of the thermal properties is important because energy conversion and dissipation are critical in real-world applications. Fig. 8(b and e) shows the temperature dependence of the entropy and temperature product (TS) and enthalpy (H), respectively, which provides information on the stability of the thermodynamics and behavior of the compounds.

The relations are given by (*H* = *U* + *pV*) and (*F* = *U* − *TS*), where *V*, *p*, and *V* represent the volume of the system, pressure, and internal energy, respectively. The *TS* plots got steeper with temperature as *C*_v_ increased progressively from S- to Se-based compounds at a given temperature. In addition, Fig. 8(e) shows that the enthalpy (*H*) increases with temperature, while *TS* increases very quickly over the same temperature range. Among the studied compounds, CoSc_2_Se_4_ always had the maximum enthalpy and *TS* values, indicating relatively high thermal stability. The increase in enthalpy was due to increased thermal motion with increasing temperature, which led to increased internal energy and the pressure-volume term in *H*.

**Fig. 9:**
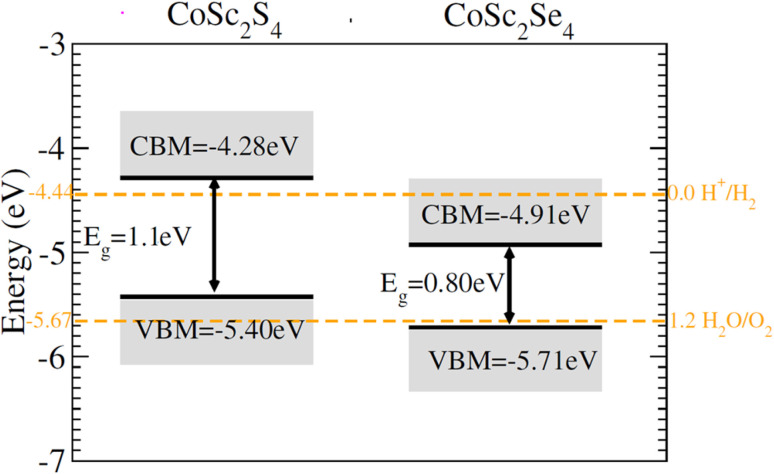
Band alignment for the CoSc_2_S_4_ and CoSc_2_Se_4_ spinels, displaying their photo-catalytic viability for water splitting.

Semiconductors with an appropriate band gap can be used to harvest solar energy for photocatalytic water splitting and hydrogen production as a clean and renewable energy carrier.^45^ The photo-generated electrons and holes reduce and oxidize water molecules, respectively.^46^ The photocatalytic property of CoSc-based compounds is predicted by Mulliken electronegativity (*χ*), and the band edge levels are calculated from *E*_VBM_ = *χ* − *E*_elec_ + 0.5 *E*_g_ and *E*_CBM_ = *E*_VBM_ + *E*_g_.^47,48^ In these equations, *E*_g_ is the band gap, *E*_elec_ = 4.5 eV is the standard electrode potential on the hydrogen scale, and *E*_CBM_ and *E*_VBM_ are the conduction and valence band edge levels, respectively, at pH = 0. The band edge levels of CoSc calculated from the Mulliken electronegativity are shown in Fig. 9. In this energy diagram, the standard reduction and oxidation potentials for overall water splitting reactions were set to −4.44 eV (referred to the Fermi level) and −5.64 eV, respectively.

The CB and VB band edges are referenced to the standard hydrogen electrode by setting the Fermi level to −4.44 eV.^45^ At pH = 0, the CB and VB edges for the overall water splitting reaction are set to 0 eV and 1.23 eV, which translate to −4.44 eV and −5.67 eV on the absolute scale, respectively.^44^ The CB and VB edge potentials of the CoSc-based compounds were calculated using the mBJ + GGA approach, and the band alignments satisfied the general redox window for the overall photocatalytic water splitting reaction at pH = 0. For efficient overall water decomposition, the conduction band minimum (CBM) should be more negative than the hydrogen reduction potential (H^+^/H_2_), while the valence band maximum (VBM) should be more positive than the oxygen evolution potential (H_2_O/O_2_). For the CoSc_2_S_4_ spinel, CBM lied slightly above the reduction potential, favoring hydrogen evolution, whereas VBM was close to the oxidation level, indicating partial suitability for water oxidation. In CoSc_2_Se_4_, VBM satisfied the oxidation criterion more effectively, although CBM was comparatively less favorable for hydrogen reduction. These results suggest that the materials are promising photocatalysts for large-scale solar-driven hydrogen evolution.

## 4. Conclusion

This was a density functional theory (DFT + mBJ)-based study to investigate the magnetic, mechanical, and optoelectronic properties of CoSc_2_(S/Se)_4_ chalcogenide spinels, which crystallised in the cubic *Fd*3̄*m* space group. The calculated second-order elastic stiffness constants satisfied the Born stability criteria for cubic crystals, thereby confirming the mechanical stability of these compounds. The elastic moduli that were taken out also showed that Poisson's ratio was higher than the critical value of 0.26 and Pugh's ratio (*B*/*G*) was higher than 1.75. Both of these are well-known signs of ductile behaviour. This indicated that CoSc_2_(S/Se)_4_ could bend a lot before it broke. The compounds were thermodynamically stable because they had negative formation enthalpies of −1.61 eV for CoSc_2_S_4_ and −1.39 eV for CoSc_2_Se_4_. We found direct band gaps of 1.1 eV and 0.8 eV (spin-up configuration) using GGA + mBJ, which indicated that the compounds were semiconductors in nature. This was in accordance with the electronic band structure and density of states results. Optical absorption spectra showed that the compounds were good for solar cell applications because they could absorb light efficiently within the visible range. The total magnetic moment suggested that both compounds were ferromagnetic, probably due to the presence of Co ions. The results concerning thermoelectric properties showed large Seebeck coefficients ranging from 248 to 240 µV K^−1^, thereby exhibiting a good figure of merit. All these clearly suggested that the compounds could be used in thermoelectric devices too.

## Conflicts of interest

There are no conflicts to declare.

## Data Availability

The data generated during the study is available from the corresponding author upon reasonable request.
